# Immune Aging and How It Works for Inflammation and Fibrosis

**DOI:** 10.3389/fphys.2021.795508

**Published:** 2022-01-04

**Authors:** Hiroshi Nishiura, Mai Imasaka, Koji Yamanegi, Jiro Fujimoto, Masaki Ohmuraya

**Affiliations:** ^1^Department of Pathology, Hyogo College of Medicine, Nishinomiya, Japan; ^2^Department of Genetics, Hyogo College of Medicine, Nishinomiya, Japan; ^3^Department of Surgery, Hyogo College of Medicine, Nishinomiya, Japan

**Keywords:** aging, immune system, inflammation, niche, stem cell

## Abstract

Almost all mature cells that undergo apoptosis in an age-dependent or an accidental manner are completely recovered in tissue-specific microenvironments without any physiological changes. After peripheral blood leukocytes are released into the local region, fibroblast cells and new blood vessels commonly proliferate during wound healing. Inducible repair tools mainly supplied from blood vessels are cleared by peripheral blood phagocytic macrophages. Finally, hematopoietic stem cell (HSC)-derived precursor cells migrate from bone marrow (BM) to the microenvironment to rebuild damaged tissues (the mature immune system). In contrast to the mature immune system, the effects of aging on HSCs (long-term HSCs) and peripheral blood lymphocytes (long-term PBLs) are not clearly understood in the BM and thymus niches with tissue-specific microenvironments with some physiological changes (the aged BM niche) for incomplete rebuilding of damaged tissues (the aged immune system). In this review, the roles of the aged immune system in both a delay of acute inflammation and the development of chronic inflammation or fibrosis are discussed.

## Maintenance of HSCs in the Mature BM Niche

**Figure 1:**
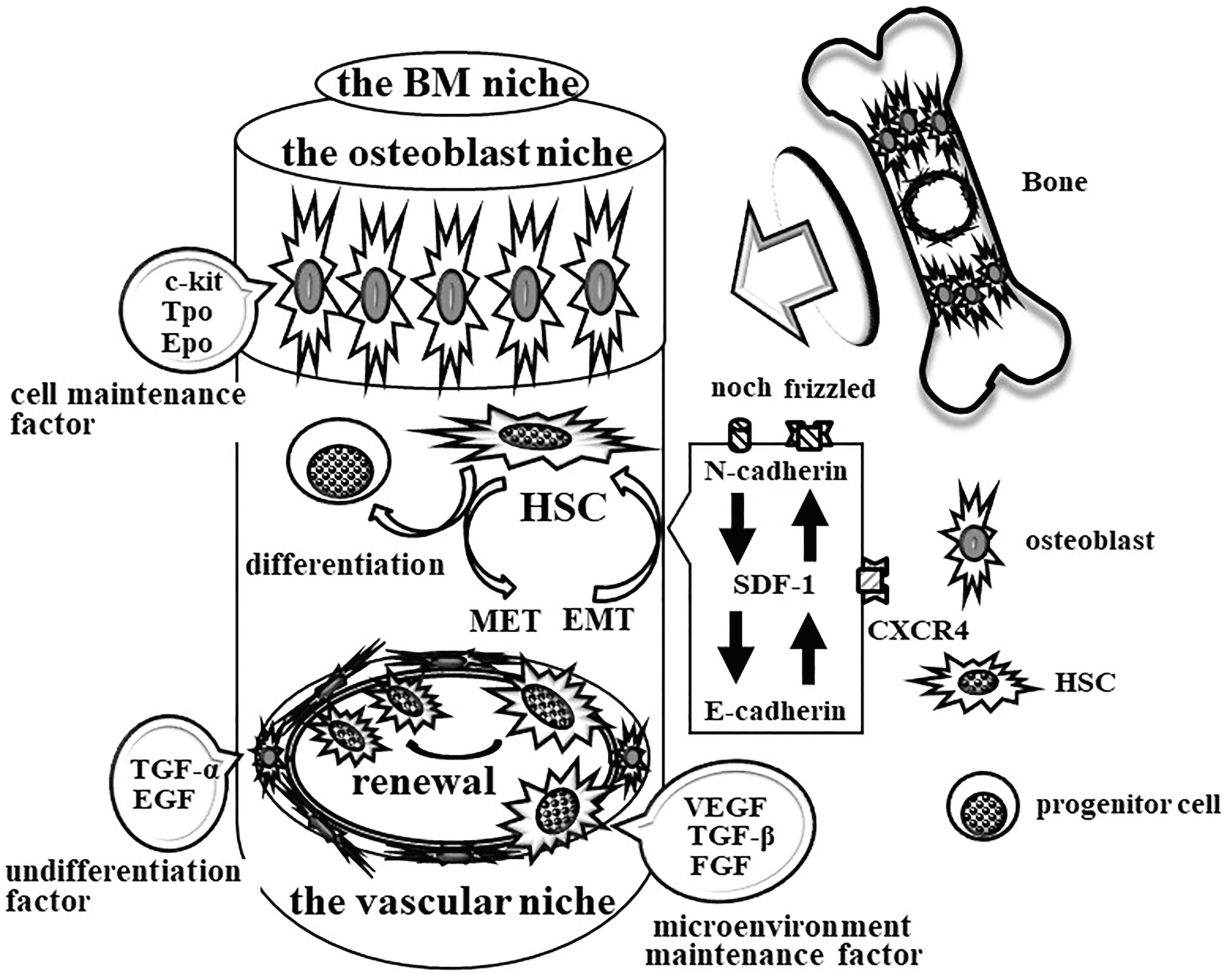
Schematic image of the maintenance system for HSCs in bone marrow (BM). The mature BM maintains the vascular niche at least by vascular endothelial growth factor (VEGF), tumor growth factor beta (TGFβ), and fibroblast growth factor (FGF). Conversely, TGF-α and epithelial growth factor (EGF) keep the nature of HSCs. E-cadherin participates in the HSC-vascular niche interaction to maintain renewal and multipotency and block differentiation in HSCs. In addition, the mature BM also maintains the osteoblast niche by c-Kit ligand, Tpo, and Epo for the maintenance of HSCs and progenitors. SDF-1/CXCL12 attracts HSCs and progenitor cells *via* CXCR4 between the vascular niche and the osteoblast niche. N-cadherin participates in the interaction of HSCs with thrombopoietin-producing osteoblasts to regulate EMT/MET.

The somatic mesoderm leads to the production of both the dorsal aspect of the aorta and hematopoietic tissue *via* vascular neonatal factors, such as vascular endothelial growth factor (VEGF), tumor growth factor beta (TGFβ), and fibroblast growth factor (FGF) in an autocrine and/or paracrine manner (Orkin and Zon, 2008). On the other hand, similar vascular neonatal factors, such as TGFα and epithelial growth factor (EGF), suppress the differentiation of HSCs, which have a Lin^lo^/c-Kit^+^/Sca-1^+^/Flt3^−^ phenotype, into mature cells in addition to the vascular niche (Pardanaud and Dieterlen-Lievre, 1999). Wnt/β-catenin signaling in HSCs plays an important role in cell homeostasis (Cobas et al., 2004; Scheller et al., 2006). E-cadherin, a transmembrane protein and major component of the adherent junction, mediates HSC-vascular niche interactions and intracellular signaling that are important to the regulation of cell behavior and organ development. Therefore, E-cadherin is an important regulator that maintains renewal and multipotency and blocks differentiation in HSCs (Gao et al., 2013). β-Catenin in conjunction with E-cadherin forms a complex with APC/Axin/glycogen synthase kinase 3 beta for ubiquitination and proteasomal degradation through the β-TrCP/Skp pathway. The recycling system constantly regulates the expression of β-catenin in the absence of Wnt signaling (Figure 1). Wnt-mediated coactivation of Notch 1 signaling is also required for homeostasis, including self-renewal of HSCs in addition to the vascular niche (Kumano et al., 2003; Duncan et al., 2005). Except for the Wnt and Notch pathways, the angiopoietin-induced Tie2-mediated pathway is involved in the expansion of HSCs (Zhang et al., 2006; Kim et al., 2017). Tie2 commonly promotes endothelial cell proliferation *via* GRB2/Ras/Raf/MEK/extracellular signal-regulated kinase (ERK) phosphorylation or cell survival and cell–cell interaction *via* phosphatidylinositol 3-kinase (PI3K)/Akt phosphorylation. The prostaglandin-induced G protein-coupled receptor (GPCR) family EP4 receptor-mediated pathway is involved in augmenting HSC production (North et al., 2007; Villablanca et al., 2007). The EP4 receptor commonly contributes vasodilation *via* cAMP/protein kinase A phosphorylation and focal cell adhesion *via* cAMP/Rap1 phosphorylation.

The other vascular neonatal factor, stromal cell-derived factor 1 (SDF-1/CXCL12), attracts HSCs and progenitor cells *via* the chemotactic GPCR family’s C-X-C chemokine receptor type 4 (CXCR4) between the vascular niche and the osteoblast niche in BM. Migrating HSCs arrive at osteoblasts under the regulation of bone morphogenetic protein (Sugiyama et al., 2006). N-cadherin mediates the interaction of HSCs with thrombopoietin-producing osteoblasts and intracellular signaling that are important to the regulation of epithelial-mesenchymal transition (EMT; Zhang et al., 2003; Hosokawa et al., 2007; Kiel et al., 2007). The BM space also contains stromal cells that support hematopoiesis and the production of cytokines, such as c-Kit ligand, thrombopoietin (Tpo), and erythropoietin (Epo), that maintain HSCs and progenitors.

## Maintenance of Long-Term HSCs in the Aged BM Niche

The BM environment may be regulated even to initiate autoimmune diseases, such as rheumatoid arthritis (RA; Conboy and Rando, 2012). The nature of HSCs in the BM of RA patients seems to change with survival, proliferation, and aging. Therefore, the nature of the components of the BM niche is also of interest. RA-like disease spontaneously developed in Balb/C interleukin-1 receptor antagonists in knockout mice by 20 weeks. Before progression of RA-like disease, small numbers of mesenchymal progenitors are found with adipogenesis, which is associated with a decrease in both osteoclastogenesis and osteoblastogenesis. Therefore, a relationship may exist between the progression of RA-like disease and the potential for self-renewal and multilineage differentiation of HSCs, which might be changed due to the nature of the BM niche in the BM environment of RA patients.

The change in the nature of the BM niche also affects immunosenescence and proinflammation *via* the regulation of age-associated changes in HSCs (Van Zant and Liang, 2012). There is a major question as to whether some kinds of HSC transplantation for cancer or gene therapies are less effective in elderly patients than in adult patients. The expansion of transplanted HSCs likely depends on the BM environment of patients. Further study of factors for maintenance not only of the HSC nature but also of the BM niche environment is necessary to increase the success rate of transplantation.

According to HSC aging, some histone modifications and DNA methylation have been found in the chromatin structure of a tissue-specific subset of genes among many tissues (DeCarolis et al., 2015). Age-related changes, such as trimethylation of lysine 4 at histone H3 and DNA methylation, specifically introduce age-related phenotypes (ARPs) of cells. However, age-related genetic changes are found in almost all proliferating cells but not in quiescent stem cells. In contrast, epigenetic modifications are found in HSCs, occasionally inducing cell heterogeneity and resulting in homing HSCs to niches. The homing of HSCs to the niche is retarded by epigenetic aging. Conversely, ARPs also retard the aging of the entire HSC population *via* the induction of HSC renewal.

It is known that a combination of mesenchymal stem cells (MSCs) and decellularized extracellular matrix (DECM) is the classical strategy for repairing tissue injury (Kovtonyuk et al., 2016). A recent paper demonstrated that DECM specially prepared from synovium-derived SCs (SDSCs) of fetal donors could help to rejuvenate human mature SDSCs, which had increased potency for proliferation and chondrogenesis. Mature DECM seems to be able to maintain the characteristics of MSCs. Further study is necessary to show the effects of age on changes in the physical properties and chemical composition of ECM.

In the steady state, HSCs are mostly quiescent, while hematopoietic progenitor cells (HPCs) actively proliferate and contribute to daily hematopoiesis. Growth and aging can induce HSCs to proliferate and engage in blood formation (Kovtonyuk et al., 2016). Insulin-like growth 2 (IGF2) is highly expressed at the embryo phase in all sites where HSCs successively expand. IGF2 expression is decreased in an age-dependent manner, where HSCs home the BM niche and again become quiescent. IGF2 activates osteoblast differentiation *via* mTORC2-Akt signaling (Shi et al., 2015). The expression level of IGF2 seems to regulate the interaction between HSCs and the BM niches through maintenance of BM formation. IGF2 most likely plays a part in the maintenance of the balance between HSC self-renewal and differentiation.

Aging-associated phenotypes of HSCs have been discussed previously, and it was reported that long-term HSCs in addition to the aged BM niche mainly contribute to aging-associated immune remodeling (Moehrle and Geiger, 2016). Immune cells devoid of DNA repair factors can usually explain the presence of aging-associated phenotypes of HSCs in the DNA damage theory. Aging-associated changes were also found in epigenetics, splicing factors, or 3D architecture in immune cells. Additionally, the aging-associated phenotypes of vascular, osteoblast, and adipocyte niches are also known. Therefore, the interaction between long-term HSCs and the aged vascular niche in at least the extramedullary hematopoiesis organs, such as the liver and spleen, must first be examined.

## Maintenance of Long-Term HSCs in the Aged Adipose Niche in BM

Adipocyte proliferation is commonly observed in the aged BM niche with a tissue-specific microenvironment with some physiological changes (Tang et al., 2008). However, the mechanism of adipocyte proliferation is still unclear. Recently, white adipocyte progenitor cells were found in addition to the tissue-specific vasculature (the adipose niche) and demonstrated participation in a differentiation mechanism. Moreover, white adipocyte progenitor cell subsets (Lin^−^/CD29^+^/CD34^+^/Sca-1^+^/CD24^+^) were shown to reconstitute normal white adipose tissue. Therefore, Lin^−^/CD29^+^/CD34^+^/Sca-1^+^/CD24^+^ subsets could rescue the diabetic phenotype of A-Zip lipodystrophic mice *in vivo* (Rodeheffer et al., 2008). These subsets have a limited potency to proliferate into adipose depots. These reports indicate a limited potency of long-term HSCs in at least the aged adipose niche.

## LNSCS Work in Mature Antigens Presenting in Acute Inflammation

**Figure 2:**
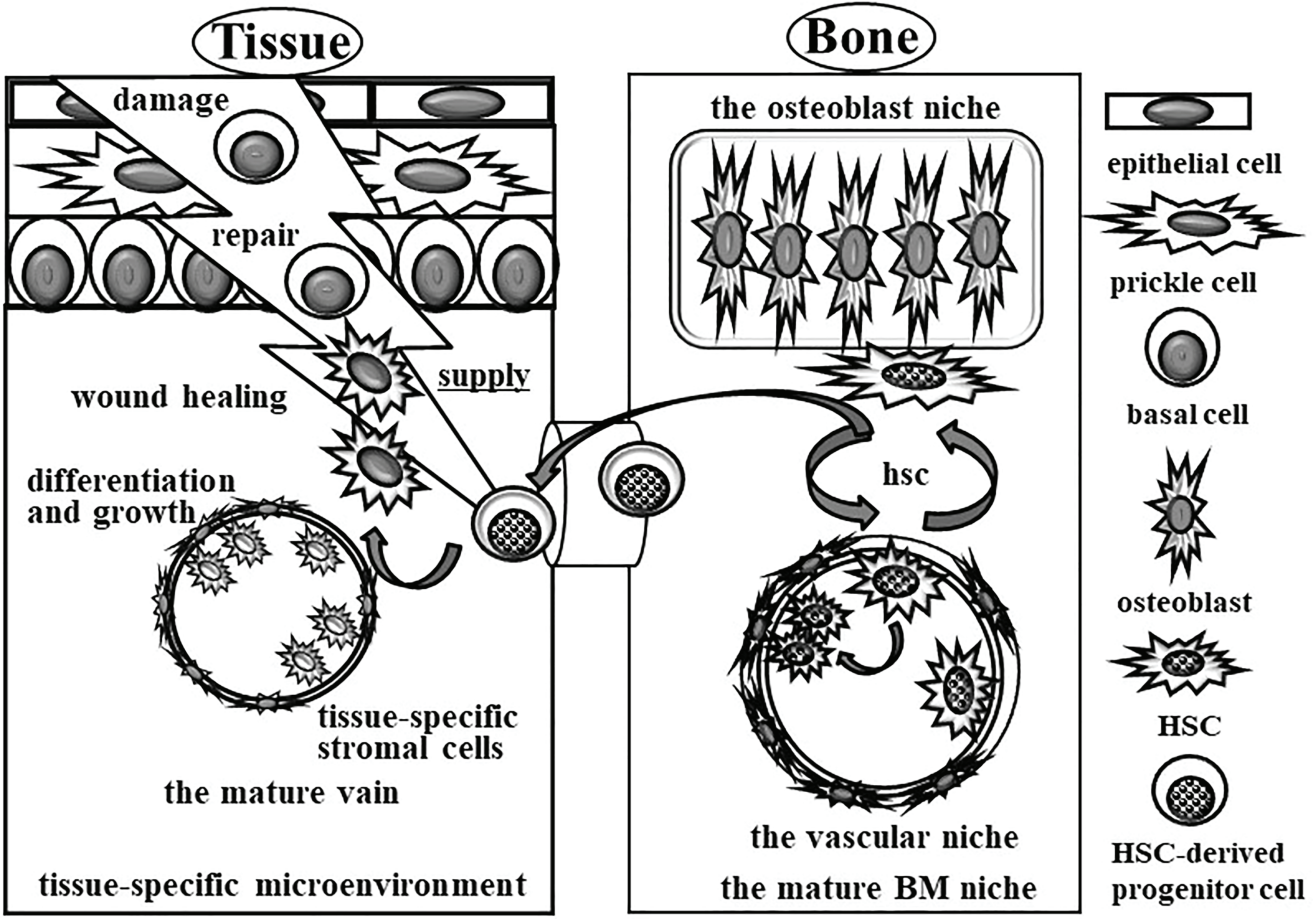
Schematic image of complete wound healing for acute inflammation by the mature immune system without changing the tissue-specific microenvironment. Acute inflammation is rescued by the mature immune system without changing the microenvironment. The repair tools are supplied from blood vessels. After treatment of acute inflammation cued by immune cells, all of the repair tools, including immune cells, are cleared by phagocytic macrophages. After that, the HSC-derived precursor cells from BM migrate into the acute inflammation site to rebuild the damaged tissues.

Antigen-induced acute inflammation is rescued by the mature immune system without changing the microenvironment (Figure 2). The repair tools are mainly supplied from blood vessels. The volume of repair tools might be regulated by the antigen-induced activation of vascular permeability. After infiltrating immune cells, fibroblast cells and new blood vessels proliferate during wound healing. Almost all antigens, including repair tools, are cleared by phagocytic macrophages. Small amounts of antigens are drained through tissue-specific lymphatic vessels into neighboring lymph nodes. Foreign antigens in small amounts of antigens in neighboring lymph nodes are selectively present on dendritic (DC) cells as MHC Class I or II for activation of CD8^+^ cytotoxic T (Th1) cells or CD4^+^ helper T (Th2) cells and B cells. The interaction among DCs, Th2 cells, and B cells enhances antigen-specific antibodies in addition to lymph node stromal cells (LNSCs), which work as alternative niches.

Recently, subclasses of LNSCs have been distinguished based on the surface expression of the glycoprotein CD31 and podoplanin (gp38). For example, fibroblastic reticular cells (FRCs), follicular dendritic cells (FDCs), lymphatic endothelial cells (LECs), blood endothelial cells (BECs), and integrinα7+ pericytes (IAPs) are classified as gp38^+^/CD31^−^, gp38^+/−^/CD31^−^, gp38^+^/CD31^+^, gp38^−^/CD31^+^, and gp38^−^/CD31^−^/ITGA7^+^, respectively. We need further examinations of disease-specific LNSCs not only in central bone marrow (BM) but also in peripheral damaged tissues or neighboring lymph nodes to understand how to work on the mature immune host defense system during acute inflammation.

## Roles of Senescent Macrophages in KI Mice in the Experimental Shift From Acute Inflammation to Chronic Inflammation

Chronic inflammation at the synovium in rheumatoid arthritis (RA) patients is caused by a predominant accumulation of monocytes/macrophages (Nishiura et al., 1996). However, the specific attraction mechanism is still unclear. We recently found a monocyte-specific chemoattractant ribosomal protein S19 (RP S19) polymer in rheumatoid arthritis-synovial tissues as an alternative ligand of the C5a receptor (C5aR) that belongs to the chemotactic G protein-coupled receptor (GPCR) family. We previously discovered a plasma-derived monocyte-specific chemoattractant that was produced by the activation of coagulation type factor XIII (FXIIIa). Regarding how macrophages predominantly migrate into coagulation clots, the cross-linked molecule in coagulation clots is most likely the RP S19 polymer. This hypothesis suggests the structure of the RP S19 polymer, which is an intermolecular cross-linkage between 122lysine (Lys: K) and 137glutamine (Gln: Q) by FXIIIa in plasma (Horino et al., 1998; Nishimura et al., 2001; Semba et al., 2010). In addition, RP S19 on protein-producing organ ribosomes is released in apoptotic cells and moves to phosphatidyl serine on the plasma membrane. RP S19 monomers are intermolecularly cross-linked by an increase in the activation of tissue type transglutaminase 2 (TG2) during programmed cell death. Moreover, we found the expression of C5aR on apoptotic cells. RP S19 polymer has C5aR binding (L_131_DR), membrane penetration (I_134_AGQVAAAN), and cell activation (K_143_KH) moieties (Shibuya et al., 2001; Shrestha et al., 2003; Nishiura et al., 2014). One L_131_DR in the RP S19 polymer released from apoptotic cells binds to apoptotic C5aR, and one K_143_KH promotes the process of programmed cell death through the activation of the apoptosis-enhancing transcription factor delta-type lactoferrin (Nishiura et al., 2015). Another L_131_DR in the RP S19 polymer on apoptotic C5aR closely binds to macrophage C5aR as an adhesion molecule, and another K_143_KH promotes phagocytic clearance of apoptotic cells through the activation of the channel activator full-length annexin A3 (Nishiura et al., 2014).

**Figure 3:**
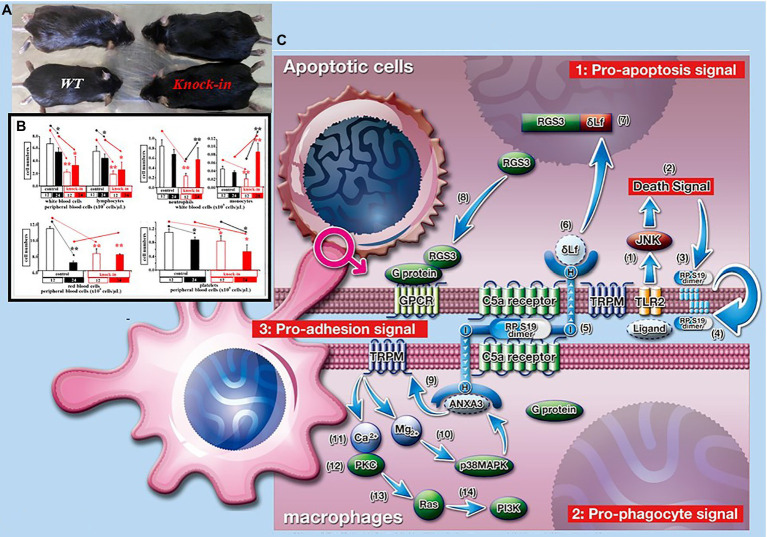
Roles of ribosomal protein S19 polymer in phagocytic clearance of apoptotic cells. **(A)** In contrast of weight of control C57BL/6J (WT) mice, that of RP S19 mutant gene knock-in (KI) mice at 24-week-olds. **(B)** In contrast of peripheral white blood cell numbers of WT mice, those of KI mice at 12-week-olds were about one to third times. At 24-week-olds, the decrement of cell number was recovered. Oppositely, peripheral red blood cell numbers were not recovered. **(C)** RP S19 polymer released from apoptotic cells arises pro-apoptosis, pro-adhesion, and pro-phagocyte signals *via* C5aRs.

Recombinant Q(CAG)137E(GAG) mutant RP S19 protein monomer was prepared by *E. coli* expression system and cross-linked by FXIIa *in vitro*. However, the Q137E mutant RP S19 polymer with alternative cross-linkages did not attract blood monocytes/macrophages. To validate this hypothesis, Q137E mutant RP S19 gene knock-in C57BL/6J mice (KI mice) were prepared (Yamanegi et al., 2017). In contrast to the number of white blood cells (WBCs) in peripheral blood cells in 12-week-old control C57BL/6J mice (WT mice), the number of WBCs slightly decreased in 24-week-old WT mice. Conversely, the number of WBCs in peripheral blood cells in 12-week-old KI mice was approximately 1/3 times that in 12-week-old WT mice (Figure 3). Interestingly, the number of WBCs in the 24-week-old KI mice was almost equal to that in the 24-week-old WT mice. Moreover, not only lymphocytes but also neutrophils and macrophages followed the same pattern as WBCs. On the other hand, the number of bone marrow cells (BMCs) in 12-week-old control C57BL/6J mice (WT mice) and the number of WBCs slightly decreased in 24-week-old WT mice. Conversely, the number of BMCs in the 12-week-old KI mice was approximately 2/3 times that in the 12-week-old WT mice. However, the number of BMCs in the 24-week-old KI mice did not recover to that in the 24-week-old WT mice. These data indicated that the number of WBCs with physiological quality was small in the 12-week-old KI mice. We suggest that the number of WBCs of nonphysiological quality is large in 24-week-old KI mice. Further examinations are needed to understand the differences in roles between WBCs of nonphysiological quality and WBCs of physiological quality.

When coagulation clots prepared from 12-week-old WT mice were injected into the abdomen of 12-week-old WT mice, the coagulation clots disappeared for at least 48 h after injection (Nishiura et al., 1996). In this experimental setting, a large number of macrophages were covered with coagulation clots. This means that macrophages in the 12-week-old WT mice physiologically phagocytose the coagulation clots. Conversely, when coagulation clots prepared from 12-week-old WT mice were injected into the abdomen of 12-week-old KI mice, the coagulation clots did not disappear for at least 48 h after injection. In this case, few macrophages were covered with the coagulation clots. This means that macrophages in 12-week-old KI mice have no physiological condition for phagocytes.

To validate the roles of RP S19 polymer in wound healing of acute inflammation *in vivo*, acute pleurisy model mice were prepared by an injection of 50 μl of 1% carrageenan into the thoracic cavity of 12-week-old WT mice (Horino et al., 1998). In this experimental setting, the neutrophils infiltrating into the thoracic cavity was almost completely cleared by macrophages for at least 24 h in 12-week-old WT mice. On the other hand, large numbers of neutrophils and macrophages were still observed at 24 h in the thoracic cavity of the KI mice after carrageenan injection. Moreover, the cells did not completely disappear at 7 days in the thoracic cavity of the KI mice after carrageenan injection. These data suggest one of the shift mechanisms of acute inflammation to chronic inflammation in young patients. Macrophages of nonphysiological quality are likely one of the key players in acute inflammation in not only old patients but also young patients. However, we did not clearly explore macrophages of nonphysiological quality.

## Roles of Senescence Lymphocytes in Inflammation

We do not completely understand the state of immune exhaustion in acute inflammation, especially the mechanism regulating T-cell receptor (TCR)-mediated signaling to avoid recognition by self-antigens. Programmed cell death 1 (PD-1), as an immune checkpoint molecule, suppresses T-cell activity during immune responses (Shibuya et al., 2001). Transcription of PD-1 is sometimes activated by interferon (IFN), which releases several kinds of peripheral blood cells at acute inflammation sites, such as CD4^+^/CD11c^−^ type-2 dendritic cell precursors and osteoclast precursor cells (Wu et al., 2018). In this case, the activation time is limited to approximately 7 days. Conversely, TCR activation by lipopolysaccharide between CD4^+^ helper T (Th2) cells and antigen presenting cells (APCs) markedly increases PD-1 ligand 1 (PD-L1) expression on macrophages *via* an induction of IFNγ. PD-L1^High+^ macrophage phenotypes suppress TCR-mediated signals (Kumano et al., 2003). Therefore, we have a new mechanism by which Th2 cells may regulate the state of immune exhaustion in acute inflammation. An interesting report found that the nature of lymphocytes in chronic tonsillitis is different from that in a peritonsillar abscess as acute severe tonsillitis or tonsil hyperplasia (Geissler et al., 2017). Further examinations are needed to classify PD-1^+^ lymphocyte subtypes based on personal characteristics, age, disease, tissue, and so on.

## Roles of Senescent Lymphocytes in Old Patients

We know that acute inflammation and chronic inflammation are not completely rescued in older patients. However, the nature of the aged immune system and subsequent physiological changes in the microenvironment depend on a personal predisposition to disease. Therefore, we need sequence and individual data from personal patients at old age to select appropriate drugs.

One of the important endogenicities for maintenance of the aged immune system is physiological atrophy of both the BM and the thymus. This change gives us a small number of newborn white blood cells. On the other hand, incomplete wound healing in old patients does not cue blood stem cells in BM to differentiate into mature immune cells. However, WBC numbers in elderly people are not significantly different from those in young people. In the aged immune system, differentiated lymphocytes with PD-1 show increased differentiation potency. Moreover, a slow cell cycle or an anti-apoptotic potency of lymphocytes was demonstrated. Currently, we have a senescence cell marker, such as H2A.X Variant Histone (H2AX) for DNA damage response (DDR); p16ink4a, p21, and p53 for cell cycle regulation; senescence-associated beta-galactosidase (SA-β-gal) for lysosome-related protein; and IL-6, IL-8, and VEGF for senescence-associated secretion protein (SASP). SASP induces a chronic-like microenvironment in legion. Further examinations are needed to determine the roles of senescent lymphocytes in alternative differentiation potency in older patients.

## Niche-Like Structures in the Secondary Lymphatic Organs in Old Patients

There are likely vascular niche-like structures and peripheral nerves with a myelin sheet at the secondary lymphatic organs. Podoplanin/gp38, a small cell-surface mucin-like glycoprotein, participates in the development of the alveoli, heart, and lymphatic vascular system (Eckert et al., 2016). Gp38 expression is upregulated not only in fibroblasts or epithelial cells but also in macrophages or Th cells during inflammation. Gp38 interaction with the same cell or in neighboring cells leads to the regulation of proliferation, migration, EMT, and remodeling of the extracellular matrix. Therefore, gp38 may participate in the prefoliation of senescent Th cells in the chronic-like microenvironment in old patients.

## Maintenance Mechanism of Liver in Old Patients

The microenvironment of liver sinusoids is maintained by cell–cell interactions among sinusoidal endothelial cells (HSECs), Küpper cells, and stellate cells (HSCs; Zhang et al., 2003). Under physiological conditions, HSCs stock vitamin A. Conversely, under inflammatory conditions, vitamin A storage is decreased and transforms into myofibroblasts through a stellate-mesenchymal transition (StMT), which is characterized by increased expression of pericellular matrix proteins, such as α-smooth muscle actin and vimentin. In addition, HSCs secrete abundant extracellular matrix proteins, such as fibronectin and collagen type I and III, *via* the production of inflammatory, proliferative, and fibrosis-related cytokines, such as interleukin-6 (IL-6), platelet-derived growth factor (PDGF), and TGFβ (Cobas et al., 2004). Therefore, we believe that the main driver of liver fibrosis is HSCs. However, the origin of HSCs is still unclear.

In experimental fibrosis progression model mice, multidrug resistance protein 2 (MDR2) ^−/−^ mice received tetrachloromethane (CCl4), which is mediated by cytochrome P450 and has the potential to lead to cancer, for 3 or 6 weeks (Duncan et al., 2005). In this model, Gp38^+^ cells were significantly expanded according to fibrosis progression. Based on the expression of the hematopoietic stem cell (HSC) marker CD133, the mature cell subsets of Gp38^+^ cells were distinguished in the CD45^−^ (leukocyte common antigen)/CD31^−^ (leukocyte common antigen)/Asgpr1^−^ (asialoglycoprotein receptor 1) liver cell fraction, such as gp38^HIGH+^ CD133^−^, gp38^LOW+^/CD133^−^, and gp38^−^/CD133^−^. The undifferentiated cell subsets of Gp38^+^ cells were also distinguished based on their gp38 expression, such as gp38^−^/CD133^+^ and CD133^+^/gp38^+^, suggesting that the distribution of the identified subsets in inflammation illustrates injury-specific changes. The most important view of this report is the existence of gp38^+^/CD133^+^ cells within the stromal/progenitor cell compartment in the liver-specific niche. Recently, Gp38^+^ cells have also been found to be present in the livers of human patients with primary biliary cirrhosis. Most likely, this is a novel pathway in chronic liver inflammation and fibrosis.

## Senescence Cells Work in Fibrosis in Liver

Cellular senescence is found not only in the normal aging process but also in several kinds of diseases. During senescence, the nature of cells is exchanged by alterations in telomere length, nuclear area, and genomic and mitochondrial DNA. As a result, senescent cells have cell cycle arrest and secrete proinflammatory cytokines (Nern and Momma, 2006). Recently, several human and animal studies have emphasized the involvement of senescent cells in the pathogenesis and development of liver steatosis, including the progression to nonalcoholic steatohepatitis (NASH), which is characterized by the additional emergence of inflammation, hepatocyte ballooning, and liver fibrosis (Scheller et al., 2006).

P16 and p53 inhibit the cyclin-cyclin-dependent kinase complex to move from the G1 period to the S period. In a p16-Cre^ERT2^-tdTomato mouse model, tdTomato-positive p16^high^ cells were found in all organs, especially increasing according to age (Sugiyama et al., 2006). These senescent cells were dominantly present in the hepatic endothelium and in renal proximal and distal tubule epithelia, respectively. They suggested that p16^high^ HSECs and Küpper cells contribute to hepatic lipidosis and immune cell infiltration. However, alternative hepatic fibrosis mechanisms other than HSCs are not clearly understood.

## CD40^+^ Cells in Liver Sinusoid Work in Fibrosis

**Figure 4:**
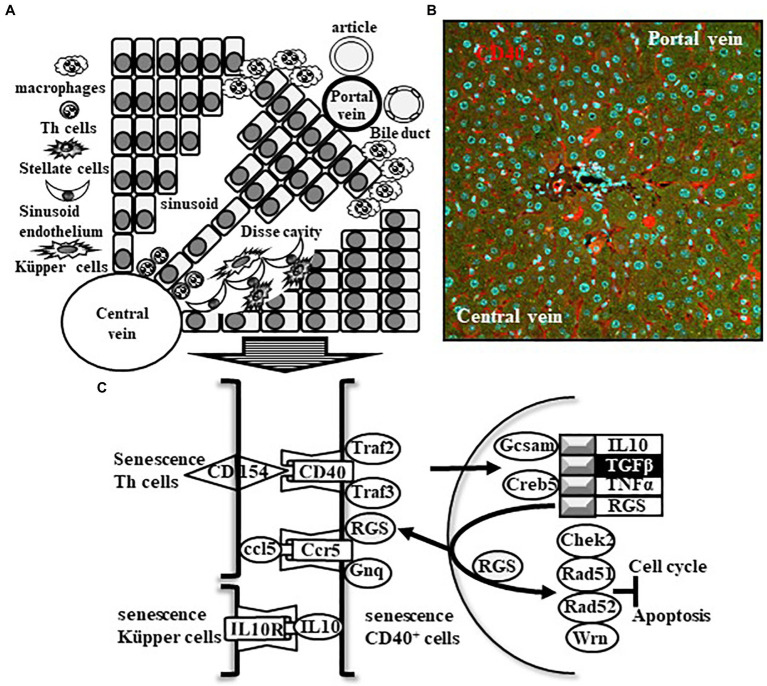
Schematic image of inflammation and fibrosis in old nonalcoholic steatohepatitis (NASH) patients. Cell–cell interactions between stellate cells, senescent sinusoid endothelium, and senescent Küpper cells at the Disse cavity of the liver sinusoid of elderly NASH patients induce the activation of HSCs for inflammation and fibrosis. **(A)** This mechanism is a main pathway. **(B,C)** Conversely, infiltrating senescent Th cells into the liver sinusoid interacts with senescent CD40^+^ cells during SASP-induced chronic inflammation in a disease-specific microenvironment, and downstream signal-dependent production of TGFβ participates in an alternative fibrosis mechanism.

HSECs significantly increase the expression of CD40 and CD80, and the latter molecule stimulates Th cells through an interaction with CD28, which is a costimulator of TCR-mediated downstream signaling. The former molecule CD40 on HSECs is inversely stimulated by the Th cell activation marker CD40 ligand CD154 upon activation of Th cells (Zhang et al., 2006). CD40 is a member of the TNF receptor superfamily, and its downstream signal is connected to the production of TGFβ (Hosokawa et al., 2007). We recently found that CD40^+^ cells were observed in the liver sinusoids of KI mice (Figure 4). However, we did not identify CD40-expressing cells in KI mice. There is an interesting study reporting that endothelial cells can transdifferentiate into mesenchymal cells through endothelial-mesenchymal transition (EndMT) and activate myofibroblasts by TGFβ (Pardali et al., 2017). We hypothesize that the cell–cell interaction between HSECs and infiltrating Th cells into the hepatic sinusoid might be an alternative fibrosis mechanism in older patients with NASH.

## CD40^+^ Cells in Pancreatitis Work in Fibrosis

Pancreatic stellate cells (PSCs) are well known to contribute with both the termination of wound healing in acute inflammation by a production of several kinds of cytokines and fibrosis in chronic inflammation by a conversion to myofibroblast which mainly expresses α-smooth muscle actin (α-SMA; Fitzner et al., 2012).

Recently, there was an interested report that was an experimental model of senescence PSCs. Primarily PSCs were prepared from pancreas of male Lewis rats in a common manner and confirmed senescence phenotype after six passages by staining with senescence-associated β-galactosidase (SA-β-Gal) which is one of the famous markers distinguished from quiescent form. They found interleukin 6 production which is a central role in fibrosis in the experimental senescence model PSCs. Moreover, SA-β-Gal-positive, α-SMA-positive, and CD4^+^ cells were found besides pancreatic duct in the dibutyltin dichloride-induced chronic pancreatitis model rats. As well as HSCs, the aging-independent and/or the aging-dependent senescence PSCs also play as the SASP to modify microenvironment for supporting organ cells including cancer cells (Moir et al., 2014).

**Figure 5:**
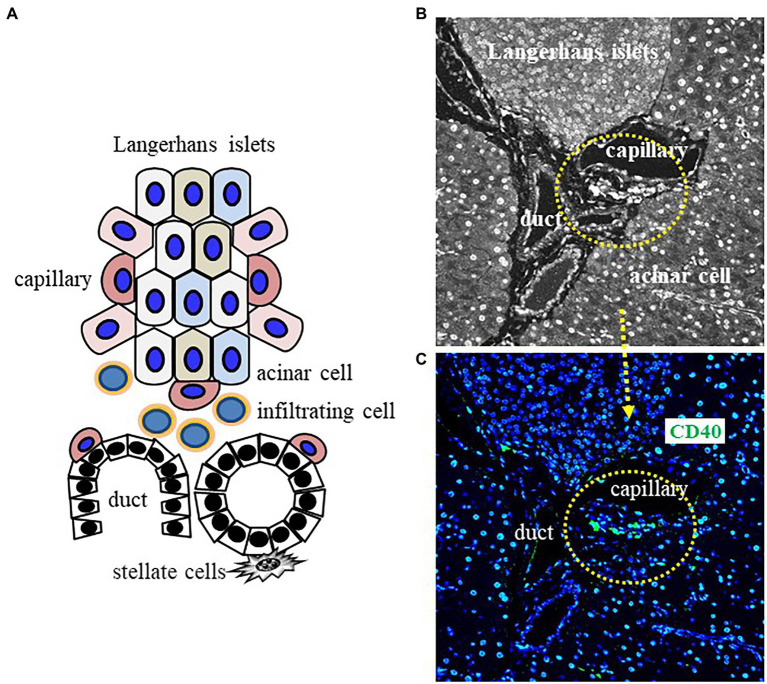
Schematic image of inflammation in old patients with pancreatitis. Cell–cell interactions between stellate cells, capillary cells, and infiltrating CD40^+^ cells beside duct of pancreas of elderly pancreatitis patients induce the activation of pancreatic stellate cells (PSCs) for chronic inflammation and fibrosis. **(A)** Interaction model of senescence cells in pancreas. **(B,C)** Infiltrating CD40^+^ cells into duct interacts with PSCs during SASP-induced chronic inflammation in a disease-specific microenvironment.

These data indicated that a contribution of CD4^+^ lymphocytes in pancreatitis with both the activation of PSCs for the initiation of wound healing and the induction of apoptosis of senescence PSCs for the termination of wound healing. We also found infiltrating CD40^+^ cells into pancreatic duct of NASH model KI mice (Figure 5). Probably, senescence CD4^+^ lymphocytes besides island of pancreas contribute with both the activation of senescence PSCs for production and release of cytokines in microenvironment and the conversion of PSCs to myofibroblasts for the initiation of anti-apoptosis effect on cancer cells. We need further examinations to support our hypothesis.

## Clinical Trial of Senolytics to Age-Related Diseases

*In vivo* experiment, we well have known accumulation of senescence cells in old mice. In fact, attenuation of physical functions, such as walking speed, muscle strength, food intake, and body weight, could be reproduced by transplantation of small number of senescence cells in young mice (Xu et al., 2018). This phenomenon was reversed by depletion of senescence cells (senolytics) in old mice. Now, we have tried several kinds of approaches to do senolytics or to suppress SASP to old patients with age-related disease (Di Micco et al., 2021).

Firstly, they focused survival pathways in senescence cells including p53/p21/serpinB2, hypoxia inducible factor-1α(HIF-1α), and B-cell/CLL lymphoma 2 (BCL-2)/BCL-XL-associated molecules. Tumor suppressor gene p53 regulates expression of cyclin-dependent kinase inhibitor p21 *via* an activation of a serin protease inhibitor serpinB2 to mediate senescence. HIF-1α up-regulates several kinds of gens including cell growth to bind CBP/p300. BCL-2/BCL-XL is one of the most famous factors preventing apoptosis. Tyrosine-kinase inhibitor Dasatinib, flavonoid group of polyphenol Quercetin, and BCL-2 inhibitor Navitoclax were developed for inhibiting above survival pathways to induce apoptosis in senescence cells without significant effects on physiologically normal function cells (2021 Meijnikman-2021-Evaluating-causality-of-cellular-se NASH). At the most warrant, point is that these apoptosis-inducing drugs cannot prevent the new generation of senescence cells. The above drugs also tried to treat cancer patients. In these cases, there were generally adverse effects, such as nausea, diarrhea, vomiting, skinrashes, and so on. It meant that these drugs are not enough to be specificity and long use.

Secondary, they focused SASP pathways in senescence cells including Rapamycin and Ruxolitinib (Thoppil and Riabowol, 2020). Rapamycin inhibits an activation of lymphocytes by suppression of interleukin 2 production through mTOR pathway. Ruxolitinib originally developed for myelofibrosis, which has gene modification in HSCs, works as cell growth inhibitor *via* specific inhibition of JAK1/JAK2 signaling. In these cases, there were generally adverse effects, such as myelosuppression.

We need other approaches to inhibit a mechanism to produce senescence cells and/or SASP for treating old patients with age-related disease (Birch and Gil, 2020). BCL-XL inhibitor DT2216 for a degradation of Von Hippel–Lindau E3 ligase reduced toxicity in platelets. Other targets are the BRD4 Inhibitor IBET762 and the histone deacetylase inhibitor Trichostatin A to interfere with signaling pathway for SASP induction. However, high dose of above drugs also has adverse effects, such as promotion of senescence and development of the SASP.

We suggest an important key to develop new types of senolytic drugs, which maintains an age-dependent physiological balance between anti-aging and senescence. Now, we recommend a regulation of senescence cell functions, especially disease-related molecules for specific promotion of fibrosis, such as CD40, without senolytics.

## Conclusion

In this review, we question how to control inflammation and fibrosis in older patients with lifestyle-related diseases, including NASH. We now propose the alternative inflammation and fibrosis pathway in CD40^+^ HSECs in hepatic sinusoids other than the main fibrosis pathway in HSCs. We suggest that HSCs are activated by BM-derived macrophages following the cell–cell interaction between senescent hepatocytes and senescent HSECs. However, the physiological condition of HSCs in a NASH-specific environment with chronic inflammation is still unclear. Therefore, it is very difficult to obtain a direct strategy for treating HSCs.

Conversely, we hypothesized that senescent CD40^+^ HSECs are activated by CD154 on infiltrating senescent Th2 cells. This activation is enhanced by the cell–cell interaction among senescent hepatocytes, senescent HSECs, and senescent Küpper cells. Recently, the removal of senescent cells (senolysis) has been proposed for the treatment of lifestyle-related diseases (Pardanaud and Dieterlen-Lievre, 1999). Kidney-type glutaminase (KGA) expression is increased according to low pH by lysosomal membrane damage. This induces an enhancement of the glutaminase 1 (GLS1) gene to maintain senescent cells. Senolysis induced by a GLS1 inhibitor rescues inflammation in lifestyle-related diseases. Therefore, we propose an alternative fibrosis pathway involving the cell–cell interaction of senescent cells in a NASH-specific environment with chronic inflammation at old age. Conversely, GLS1 controls chondrocytes for long bone growth and fracture repair (Stegen et al., 2020). Further examination is needed to determine how to control inflammation and fibrosis in older patients with lifestyle-related diseases, including NASH.

## Author Contributions

HN and MO wrote the manuscript. MI, KY, and JF contributed to the article and approved the submitted version. All authors contributed to the article and approved the submitted version.

## Funding

Grant numbers and sources of support: Hyogo College of Medicine, 2021 and the Cooperative Research Program of the Institute for Protein Research, Osaka University (CR-15-01).

## Conflict of Interest

The authors declare that the research was conducted in the absence of any commercial or financial relationships that could be construed as a potential conflict of interest.

## Publisher’s Note

All claims expressed in this article are solely those of the authors and do not necessarily represent those of their affiliated organizations, or those of the publisher, the editors and the reviewers. Any product that may be evaluated in this article, or claim that may be made by its manufacturer, is not guaranteed or endorsed by the publisher.

## Figures and Tables

**Table 1:** 

ARPs	Age-related phenotypes
APC	Antigen presenting cells
BECs	Blood endothelial cells
BM	Bone marrow
C5aR	C5a Receptor
Th2	CD^4+^ Helper T
Th1	CD^8+^ Cytotoxic T
FXIII	Coagulation type factor XIII
CXCR4	C-X-C Chemokine receptor type 4
DECM	Decellularized extracellular matrix
DC	Dendritic cells
DDR	DNA damage response
EGF	Epithelial growth factor
EMT	Epithelial-mesenchymal transition
Epo	Erythropoietin
ERK	Extracellular signal-regulated kinase
FGF	Fibroblast growth factor
FRCs	Fibroblastic reticular cells
FDCs	Follicular dendritic cells
GPCR	G Protein-coupled receptor
GLS1	Glutaminase 1
H2AX	H2A.X Variant histone
HSCs	Hematopoietic stem cells
HPCs	Hematopoietic progenitor cells
IGF2	Insulin-like growth factor 2
IAPs	Integrinα7+ pericytes
IFN	Interferon
IL-6	Interleukin-6
KGA	Kidney-type glutaminase
KI	Knock-in
LNSCs	Lymph node stromal cells
LECs	Lymphatic endothelial cells
MSCs	Mesenchymal stem cells
MDR2	Multi drug resistance protein 2
NASH	Nonalcoholic steatohepatitis
PBLs	Peripheral blood lymphocytes
PI3K	Phosphatidylinositol 3-kinase
PDGF	Platelet-derived growth factor
gp38	Podoplanin
PD-1	Programmed cell death 1
PD-L1	Programmed cell death 1 ligand 1
Senolysis	Removal of senescent cells
RA	Rheumatoid arthritis
RP S19	Ribosomal protein S19
SA-β-gal	Senescence-associated beta-galactosidase
SASP	Senescence-associated secretion protein
HSECs	Sinusoidal endothelial cells
HSCs	Stellate cells
SDF-1/CXCL12	Stromal cell-derived factor 1
SDSCs	Synovium-derived SCs
TCR	T Cell receptor
CCl4	Tetrachloromethane
Tpo	Thrombopoietin
TG2	Tissue type transglutaminase 2
TGFβ	Transforming growth factor beta
VEGF	Vascular endothelial growth factor
WBCs	White blood cells
